# Knowing left from right: asymmetric functional connectivity during resting state

**DOI:** 10.1007/s00429-017-1604-y

**Published:** 2018-01-04

**Authors:** Mathijs Raemaekers, Wouter Schellekens, Natalia Petridou, Nick F. Ramsey

**Affiliations:** 1 Brain Center Rudolf Magnus, University Medical Center Utrecht, Utrecht University, Str. 4.205, Postbus 85060, 3508 AB Utrecht, The Netherlands; 2 Department of Radiology, University Medical Center Utrecht, Utrecht University, Utrecht, the Netherlands

**Keywords:** fMRI, Connectivity, Lateralization, Language, Handedness

## Abstract

The functional organization of left and right hemispheres is different, and hemispheric asymmetries are thought to underlie variations in brain function across individuals. In this study, we assess how differences between hemispheres are reflected in Asymmetric Functional Connectivity (AFC), which provides a full description of how the brain’s connectivity structure during resting state differs from that of the same brain mirrored over the longitudinal fissure. In addition, we assess how AFC varies across subjects. Data were provided by the Human Connectome Project, including 423 resting state and combined language task fMRI data sets, and the pattern of AFC was established for all subjects. While we could quantify the symmetry of brain connectivity at 95%, significant asymmetries were observed, consisting foremost of: (1) higher correlations between language areas in the left hemisphere than between their right hemisphere homologues. (2) Higher correlations between language homologue areas in the right hemisphere and left default mode network, than between language areas in the left hemisphere and the default mode network in the right hemisphere. The extent to which subjects exhibited this pattern correlated with language lateralization and handedness. Further exploration in intersubject variation in AFC revealed several additional patterns, one involving entire hemispheres, and another correlations with limbic areas. These results show that language is an important, but not only determinant of AFC. The additional patterns of AFC require further research to be linked to specific asymmetric neuronal states or events.

**Electronic supplementary material:**

The online version of this article (10.1007/s00429-017-1604-y) contains supplementary material, which is available to authorized users.

## Introduction

For many brain functions, including the processing of sensory information or the encoding of bodily movements, the two hemispheres are an almost perfect mirror image of each other, with the left perceiving and controlling the right and vice versa. However, there is ample evidence for several cognitive modalities to mainly operate in one of the two hemispheres. Although left dominant speech production and hand preference are arguably the most important lateralized feature of the brain (Broca 1861; Knecht 2000), there are others including face perception (Yovel et al. 2008; Wilkinson et al. 2009) and spatial attention allocation (Ciçek et al. 2009) that are thought to have a bias for one of the hemispheres. Detailed knowledge of how the neuronal architecture gives rise to asymmetries in distributions of functions can help our general understanding of how functions emanate from brain mechanisms. In addition, it may eventually shed light on more fundamental questions addressing hemispheric specialization regarding human conscious experience (Gazzaniga 2000).

Furthermore, investigating intersubject variation in dissimilarity of the hemispheric architectures may be important for revealing neuronal underpinnings of behavioral differences across subjects. While the theory of left and right brain personality should be regarded with considerable skepticism (Nielsen et al. 2013), this does not refute the existence and relevance of other variations in patterns of brain asymmetry across individuals, that relate to specific behavioral, physiological, or personality features. For instance, depression has been linked to an imbalance in activity between the hemispheres (Henriques and Davidson 1991; Flor-Henry et al. 2004; Nielsen et al. 2013), while incomplete lateralization is thought to be one of the neuronal abnormalities underlying schizophrenia (Stephane et al. 2001; Frith 2005).

Advanced neuroimaging techniques are now available that allow for detailed descriptions of the brain’s connectivity in terms of asymmetries and hemispheric specialization (Hervé et al. 2013). In this study, we investigate the neuronal underpinnings of lateralization of brain functions and intersubject variation therein by mapping hemispheric asymmetries of the connectivity structure. For investigating these asymmetries, we chose fMRI Resting-State (RS) activation, the spatiotemporally linked spontaneous fluctuations in the hemodynamic response in the absence of sensory input or motor activity (Biswal et al. 1997; Raichle and Mintun 2006; Friston 2011). While we acknowledge uncertainty in what kind of neuronal processes these fluctuations correspond to, and that correlations in RS fluctuations do not necessarily correspond to anatomical connectivity (Sporns 2011), they do contain important signatures of the underlying connectivity (Skudlarski et al. 2008; Raemaekers et al. 2014). Furthermore, fMRI RS is relatively easy to acquire and share, and it addresses the whole brain instead of tapping into specific functions or areas.

So far, fMRI RS studies have suggested a highly symmetric connectivity, with networks as detected with, for instance, independent component analysis being identically distributed across the two hemispheres (Damoiseaux et al. 2006; Smith et al. 2009). Although two frontoparietal networks have been consistently detected that each reside in one hemisphere only, they seem to be each other’s mirror image. While they exhibit different features with respect to their activity, the connectivity strengths within their respective hemispheres may thus be indistinguishable. Any asymmetries are, therefore, prone to be relatively minute. A direct quantification of the extent of the hemispheric symmetry is, however, missing. The first objective of this study is to measure the level of asymmetric connectivity by estimating how well the brain’s connectivity structure can be predicted by the connectivity structure of the brain mirrored over the longitudinal fissure.

Recent investigations have demonstrated the existence of hemispheric asymmetries in resting-state connectivity, and that the intersubject variation therein is related to language lateralization and handedness (Wang et al. 2014; Joliot et al. 2016). While asymmetries most likely involve the frontoparietal, attention, and default mode networks, no study so far has precisely mapped the exact underlying connectivity asymmetries in full. Studies so far have either focused on asymmetries of connections within the left hemisphere or the right hemisphere (Tzourio-Mazoyer et al. 2015), address specific regions of interest (Fox et al. 2006; Xiao et al. 2016; Hasler et al. 2017), or have obtained condensed asymmetry metrics by quantifying the mean amount of asymmetric connections per voxel (Joliot et al. 2016). While these approaches certainly have their merits, they do not provide a full and simultaneously assessment of any connectivity asymmetries between and within hemispheres. This endeavor is necessary to obtain a comprehensive overview on which connections might be underlying lateralized brain functions. In addition to the full assessment of asymmetries in individual connections, we apply a data driven approach to establish if there are other sources besides language lateralization that are driving individual differences in the observed asymmetries. As we anticipate small effect sizes regarding asymmetries, data of prolonged RS measurements are required in large groups of healthy volunteers, which are made available by the Human Connectome Project (HCP) (Van Essen et al. 2012b). The need for prolonged measurements also means that at this stage, we are disregarding potential dynamics in asymmetry. As metric we use Asymmetric Functional Connectivity (AFC), which we define as the extent to which the RS correlation matrices differ from those of the same brains mirrored along the longitudinal fissure. This represents a whole-brain survey of asymmetries in inter and intrahemispheric connectivity. As this metric is to a large extent explorative, we are at the current stage not employing graph-theoretical metrics. We classified patterns of asymmetry which were subsequently linked to fMRI language lateralization and a collection of behavioral features.

## Methods

### Subjects

We included data sets of the ‘500-subjects-release’ of the HCP that contained both a complete RS and language processing task fMRI acquisition. Data sets of 423 subjects met this criterion and qualified for inclusion. The group consisted of 180 males (mean age 29.0 years; SD 3.6 years) and 243 females (mean age 29.2 years; SD 3.5 years).

### Mr data description

For the analysis, we used the preprocessed volumetric functional images of the resting state and language processing task sessions that were provided by the HCP. In addition, we used the volumes containing the automatic Freesurfer parcellation and segmentation of the T1-weighted image for region-of-interest (ROI) definitions (Van Essen et al. 2012a). Data were acquired on customized Siemens 3T ‘Connectome Skyra’ scanners with a 32-channel headcoil (Glasser et al. 2013). Whole-brain gradient-echo EPI images were acquired with the following parameters: TR 720 ms; TE 33.1 ms; multiband factor 8; FA 52°; 72 slices; FOV 208 × 180 mm; resolution 2.0 mm isotropic; BW 2290 Hz/Px.

The RS data consisted of four echo planar imaging runs of 1200 volumes each, accumulating to a total RS acquisition time of just below an hour. The RS data acquisition was spread over 2 days, and on each scanning day, one run was acquired with Left–Right (LR), and the other with Right–Left (RL) as phase-encoding direction. The language processing data were acquired on a single day, and in two runs of 316 whole-brain volumes each, using the same pulse sequence as for the RS data acquisition. Again, the phase-encoding direction was reversed between runs. The task consisted of alternating story and math blocks with a mean block length of 30 s. For a more detailed description of the language processing task, see Binder et al. (2011).

Preprocessing of functional images included (1) gradient distortion correction, (2) motion correction, (3) generating a field map using two spin echo EPI images with reversed phase-encoding directions, (4) distortion correction and EPI to T1-weighted image registration, (5) intensity normalization and bias field removal. For detailed information on all the steps, see Glasser et al. (Glasser et al. 2013). Steps 1–4 were combined in a single interpolation step.

### Data analysis

**Fig. 1:**
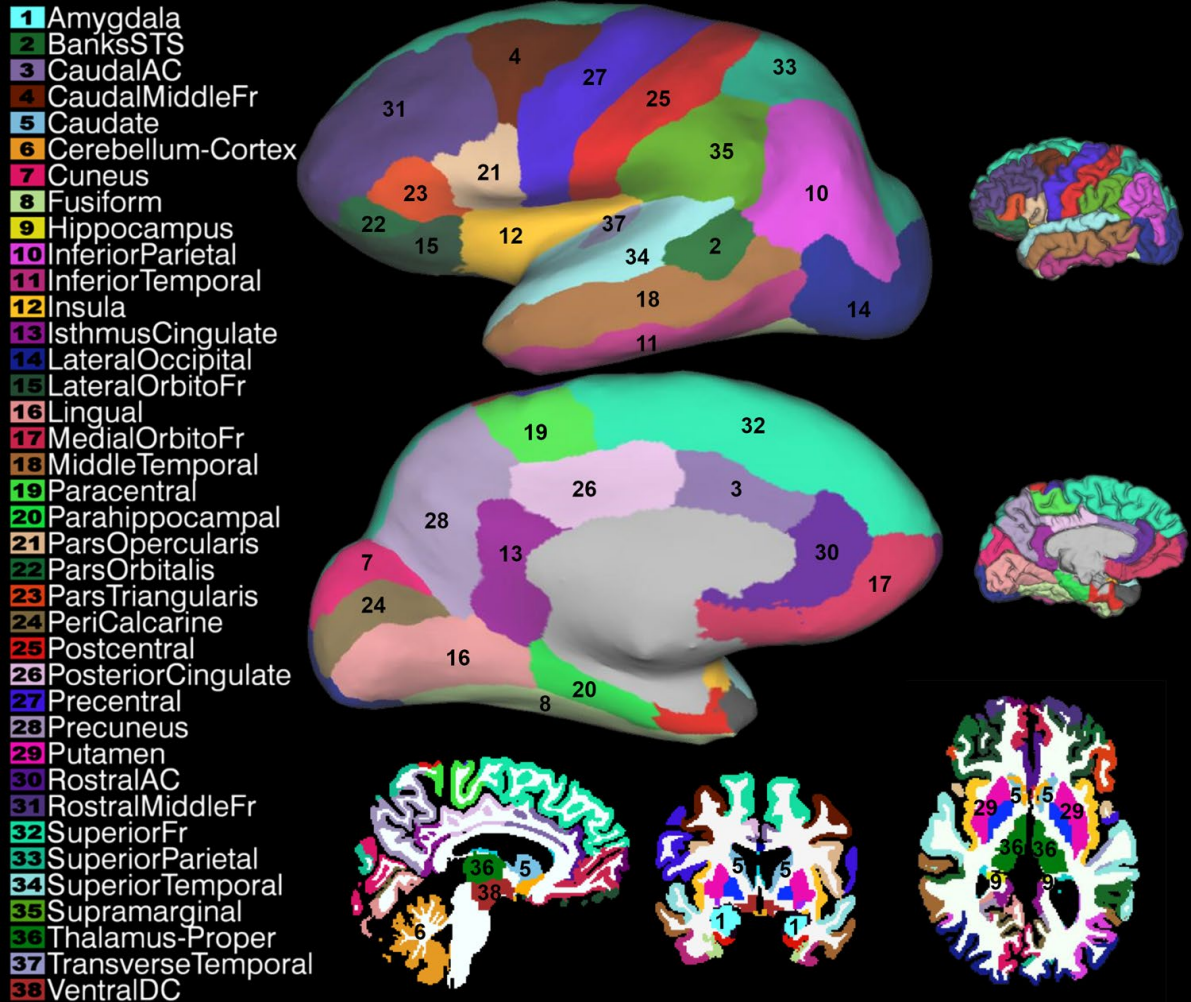
Automatic segmentation example of a single non-related subject that was scanned in our institute for reference of the ROIs used in the analysis. Different ROIs are represented by different colors and numbers on an inflated surface reconstruction of small depictions of the pial surface are added for orientation. Several volumetric slices are displayed to show subcortical ROIs

The volume containing the Freesurfer segmentation was resliced to the fMRI volumes using a nearest-neighbor algorithm in SPM12 (http://www.fil.ion.ucl.ac.uk/spm/), resulting in 76 regions of interest (ROI), which comprised 38 pairs of homologue brain areas (Fig. 1). All subsequent analysis steps were performed in IDL version 8.1 (ITT Visual Information Solutions, http://www.exelisvis.com/), using the standard IDL library for statistical operations (simple linear regression with least-squares approach, Pearson correlation, principal component analysis, and standard deviation), or custom-written scripts for the remaining basic matrix transformations.

#### Analysis of RS fMRI data

For the RS analysis, the mean timeseries were calculated for every ROI, and normalized to percent signal change for each run separately. We did not apply global signal correction as our asymmetry metric is based on the differences in connectivity between the brain and its mirrored counterpart. This means that the global signal cannot contribute to our metric unless it is expressed differently throughout the left and the right hemispheres, and in that case, the global signal would not qualify as confounder. Subsequently, all ROI timeseries-pairs were correlated, and the resulting correlation matrices were Fischer transformed to *z* values for the second-level analyses. In addition, separate correlation matrices were made for the two acquisition days (days 1 and 2) to assess intersession variability, and for the two acquisition directions (LR and RL) to assess possible confounds by asymmries in large-scale susceptibility effects due to the LR/RL acquisition. The AFC matrix was defined as follows:

$${\text{AFC}}\; - \;{\text{matrix }}=\left[ {\begin{array}{*{20}{c}} {{z_{A_{l}^{n},A_{r}^{1}}} - {z_{A_{r}^{n},A_{l}^{1}}}}&{{z_{A_{l}^{n},A_{r}^{2}}} - {z_{A_{r}^{n},A_{l}^{2}}}}& \cdots &{{z_{A_{l}^{n},A_{r}^{{n - 1}}}} - {z_{A_{r}^{n},A_{l}^{{n - 1}}}}}&\emptyset \\ \vdots & \vdots & {\mathinner{\mkern2mu\raise1pt\hbox{.}\mkern2mu \raise4pt\hbox{.}\mkern2mu\raise7pt\hbox{.}\mkern1mu}} & {\mathinner{\mkern2mu\raise1pt\hbox{.}\mkern2mu \raise4pt\hbox{.}\mkern2mu\raise7pt\hbox{.}\mkern1mu}} &{{z_{A_{l}^{{n - 1}},A_{l}^{n}}} - {z_{A_{r}^{{n - 1}},A_{r}^{n}}}} \\ {{z_{A_{l}^{3},A_{r}^{1}}} - {z_{A_{r}^{3},A_{l}^{1}}}}&{{z_{A_{l}^{3},A_{r}^{2}}} - {z_{A_{r}^{3},A_{l}^{2}}}}& {\mathinner{\mkern2mu\raise1pt\hbox{.}\mkern2mu \raise4pt\hbox{.}\mkern2mu\raise7pt\hbox{.}\mkern1mu}} & {\mathinner{\mkern2mu\raise1pt\hbox{.}\mkern2mu \raise4pt\hbox{.}\mkern2mu\raise7pt\hbox{.}\mkern1mu}} & \vdots \\ {{z_{A_{l}^{2},A_{r}^{1}}} - {z_{A_{r}^{2},A_{l}^{1}}}}&\emptyset &{{z_{A_{l}^{2},A_{l}^{3}}} - {z_{A_{r}^{2},A_{r}^{3}}}}& \cdots &{{z_{A_{l}^{2},A_{l}^{n}}} - {z_{A_{r}^{2},A_{r}^{n}}}} \\ \emptyset &{{z_{A_{l}^{1},A_{l}^{2}}} - {z_{A_{r}^{1},A_{r}^{2}}}}&{{z_{A_{l}^{1},A_{l}^{3}}} - {z_{A_{r}^{1},A_{r}^{3}}}}& \cdots &{{z_{A_{l}^{1},A_{l}^{n}}} - {z_{A_{r}^{1},A_{r}^{n}}}} \end{array}} \right].$$

**Fig. 2:**
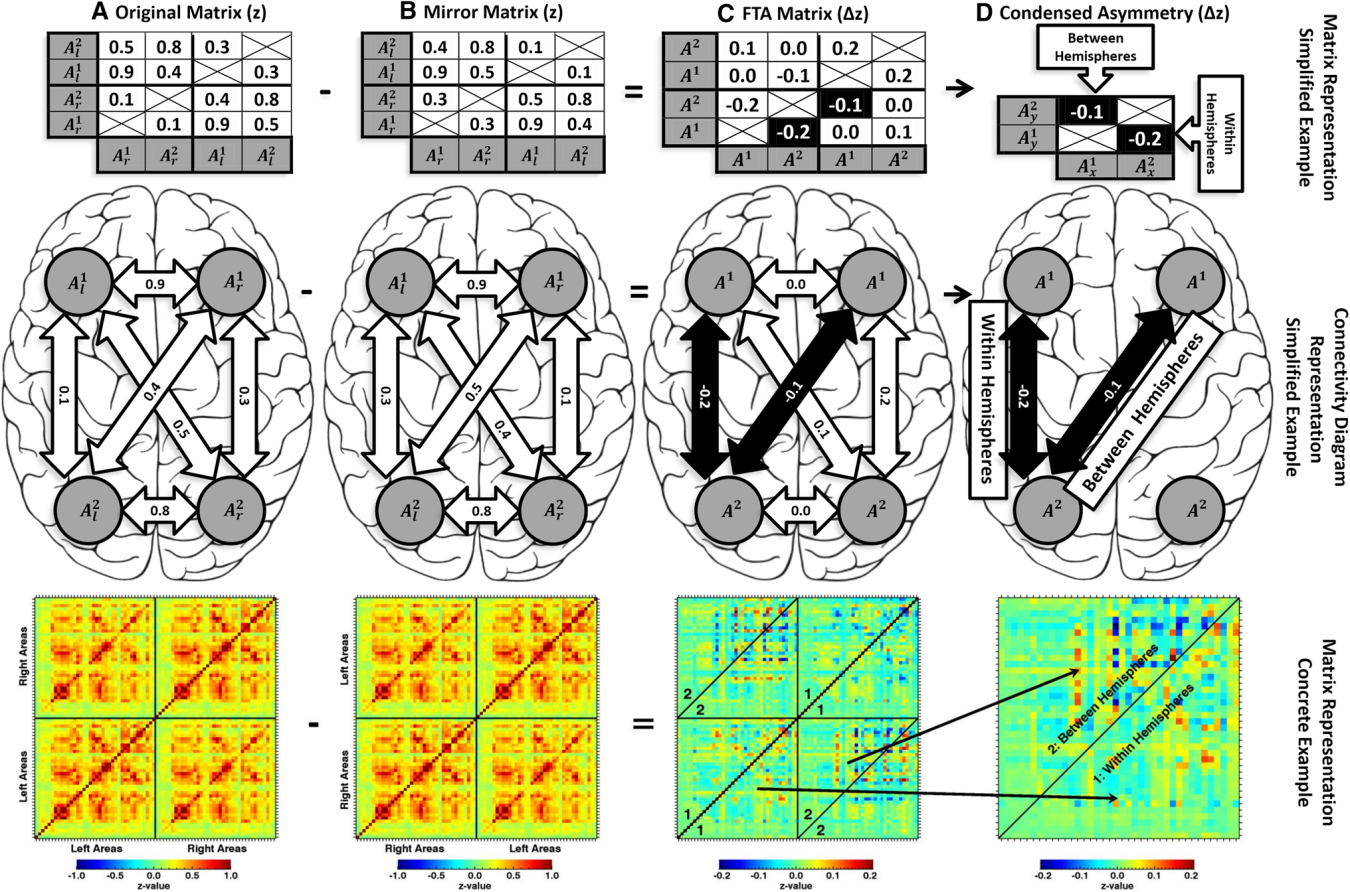
Depiction of the used approach using a simplified and a more concrete example. The simplified example involves connections between four hypothetical areas and is depicted in the first two rows, once in matrix representation (row one) and once as a brain connectivity diagram (row two). The simplified example includes left hemisphere areas 1 $(A_{l}^{1})$ and 2 $(A_{l}^{2})$ and their contralateral homologues $(A_{r}^{1}\;{\text{and}}\;A_{r}^{2})$. The concrete example is depicted in matrix representation only (row 3), and includes all the connections between all left and right brain ROIs as represented in Fig. 1. The matrices containing the *z*-transformed RS correlations between the areas is established, with the rows and columns first ordered by hemisphere, then by area (**a**). A mirror matrix is calculated by swapping left and right areas, representing the connectivity of the same brain mirrored across the longitudinal fissure (**b**). The mirror matrix is subtracted from the original RS matrix, resulting in the AFC in the RS connections. Thus, the larger the difference between the original and mirror matrix, the larger the values in the AFC matrix (**c**). Note that there is a substantial amount of superfluous data in this matrix. To condense the matrix for more compact visualization, two non-redundant portions are extracted. These portions are highlighted in black in the simplified example and subsequently represent asymmetry of within-hemisphere connections ($A_{{xl}}^{2}A_{{lyl}}^{1} - A_{{xr}}^{2}A_{{yr}}^{1}$; meaning connectivity between left *A*^2^ along the horizontal/*x* axis of the matrix, and left *A*1 along the vertical/*y* axis of the matrix, minus connectivity between right *A*^2^ along the *x* axis of the matrix, and right *A*1 along the *y* axis of the matrix), and asymmetry of between-hemisphere connections ($A_{{xr}}^{1}A_{{yl}}^{2}\; - \;A_{{xl}}^{2}A_{{yr}}^{2}$; meaning connectivity between right ${A}^{1}$ along the *x* axis of the matrix, and left *A*^2^ along the *y* axis of the matrix, minus connectivity between left *A*^1^ along the *x* axis of the matrix, and right *A*^2^ along the *y* axis of the matrix). The concrete example shows how these asymmetries of within- and between-hemisphere connections are represented by two triangular portions of the asymmetry matrix, which are combined to form the square condensed asymmetry matrix (**d**)

The elements in the matrix represent differences in *z* values for Pearson correlations between ROIs *A*^1,*n*^ in the left (l) and the right (*r*) hemispheres. Figure 2 contains a graphical depiction of how the asymmetry matrices were established, in which larger values represent larger asymmetry. Significance of group wise effects for individual correlations in the correlation matrices was assessed using one-sample and paired samples *t* tests, which were Bonferroni corrected for the number of values in the original (2850 values; as in panel 2A) and AFC matrices (1406 values; as in panel 2D). As data were acquired with the phase-encoding directions along the left–right axis. results might be confounded by large-scale susceptibility effects while testing for hemispheric asymmetries. This possibility was extensively tested and refuted (see supplement 1).

The extent of AFC per subject was established by summarizing the AFC matrix of each subject as a single value (AFC-score), which was the coefficient of a simple (one independent variable) linear regression with least-squares approach, with the individual AFC matrix as dependent, and the group-mean AFC matrix (across subjects; *n* = 423) as independent variable:

$$y=\alpha +\beta x+\varepsilon ,$$

where *y* is the vector containing the values within the individual AFC matrix, *β* is the individual AFC-score, *x* is the vector containing values within the group-mean AFC matrix, *α* is the intercept of the fit, and *ε* is the matrix with the residuals (error). The AFC-score thus represents the magnitude of the asymmetry of a subject, as defined by the amount of scaling required to optimally fit the mean group AFC matrix to the individual AFC matrix. This approach is based on the assumption that the mean AFC is exemplary of left language lateralized subjects, as the HCP group of healthy volunteers was predominantly right-handed (78% had a laterality score > 50), and right-handed subjects nearly always have left language lateralization (Knecht 2000). Thus, a high AFC-score indicates the extent to which a subject expresses the mean AFC of left language lateralized subjects. Further patterns of AFC were explored using principal component analysis (PCA), with the entries in the AFC matrix as variables, and the different subjects as the individual observations. Resulting principal component coefficients represent the patterns of asymmetry explaining most variance between subjects, and their corresponding principal component scores the extent to which these patterns are present in individual subjects. PCA was chosen instead of Independent Component Analysis to allow complete reproduction of the reported results using the same sample of the HCP, and to ascertain that observed components can be fitted to the data independently of an arbitrary number of other components in future studies.

#### Analysis of language processing fMRI data

The timeseries for each run were scaled, such that the mean value of the entire run was 100, and that changes in BOLD corresponded to % signal change. Then, the voxel timeseries were analyzed using a general linear model. The first factor in the design matrix represented increased BOLD for story vs. math. This factor was generated by convolving the boxcar function of story vs. math with the hemodynamic response function. The second factor represented the difference in intercept between the two runs. Four additional factors were added to the design matrix and consisted of cosine functions with 0.5/1./1.5/2 cycles over the two runs, that in combination formed a high pass filter with a cutoff at approximately 0.005 Hz. Regional language activity was estimated by taking the mean regressor coefficient for the story vs. math factor for every ROI. Then, these right hemisphere ROI activity estimates were subtracted from their left hemisphere homologues, resulting in a vector with a length of 38 (half the number of ROIs) that represented language lateralization. Analogously to the approach for AFC, a subjects’ Language Task Lateralization (LTL) was condensed to a single value (LTL-score), which was defined by the estimated coefficient of a simple linear regression, with the individual language lateralization vector as dependent, and the group-mean language lateralization vector as independent variable. A high LTL-score thus indicates a strong left language lateralization.

### Reliability of measures and relationship with behavior and personality

We planned to link measures of AFC to the behavioral characteristics that were assessed by the HCP, which included metrics of cognition, emotion, motor and sensory function, personality, psychiatric history, substance abuse, and physical functioning (Barch et al. 2013). We used Pearson correlations for estimating relationships between AFC variables and behavioral characteristics at and beyond ordinal scale, and F-statistics for behavioral measures at nominal scale. In addition, when linking AFC-measures to assumed stable behavioral features, it is instructive to know to what extent the acquired AFC metrics are stable features. For estimating similarity across sessions, we used the Intraclass Correlation Coefficient for absolute consistency (ICC_(2,1)_) (Shrout and Fleiss 1979). Assessments of test–retest reliability are based on half the amount of data per subject, and thus underestimate the reliability of the full measurements. Reported reliabilities are, however, informative as low-end estimates, and for comparison of reliability between AFC variables.

## Results

### Language processing task

**Fig. 3:**
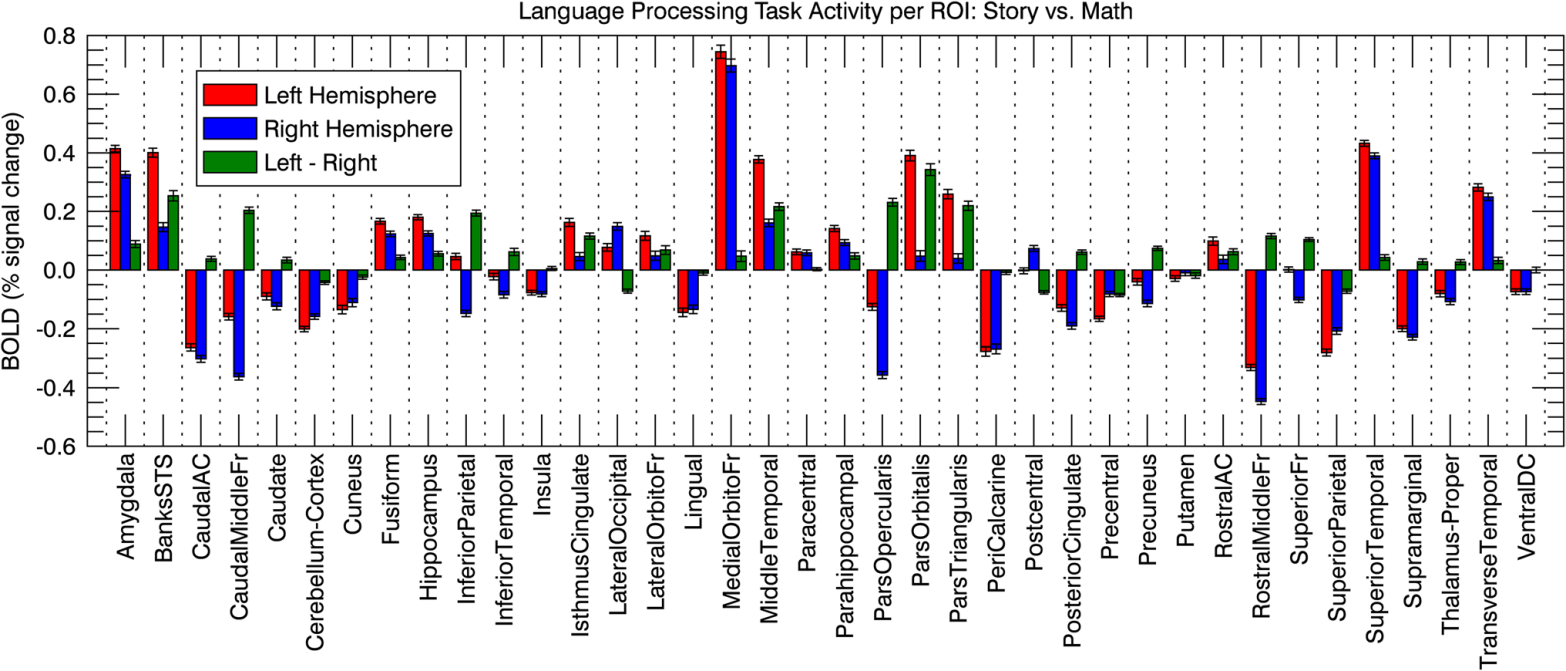
Group-mean BOLD signal change in right and left hemisphere ROIs for story vs. math during the language processing task. Green bars show the difference between left and right homologue ROIs, which in combination formed the independent variable for assessing the LTL per individual subject. Bars indicate standard errors of the mean

The language processing task showed significant lateralization effects in most ROIs, but the strongest left lateralization was in the known language areas (i.e., ParsTriangularis, ParsOpercularis, ParsOrbitalis, MiddleTemporal, BanksSTS) (Fig. 3).

**Fig. 4:**
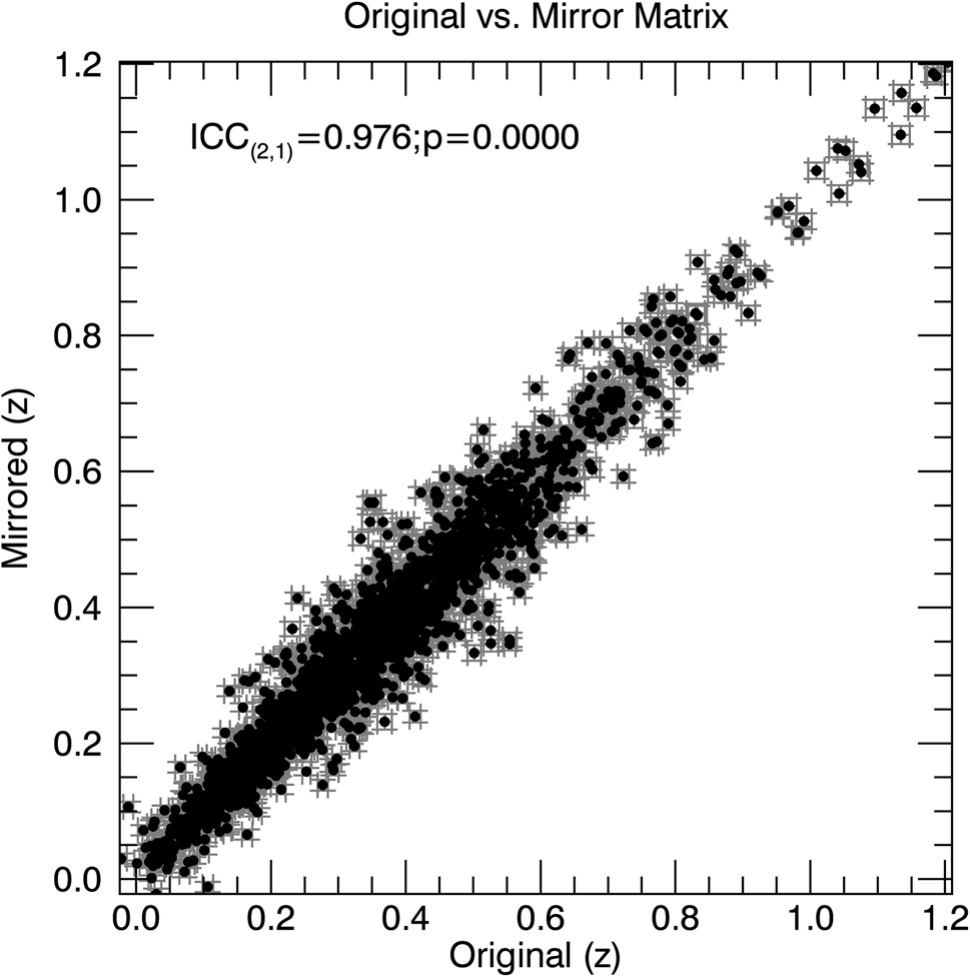
Scatterplot of the original group-mean connectivity matrix vs. the group-mean mirror matrix. Each ROI pair is represented by a single black dot, with its value within the two compared matrices represented by its *x* and *y* coordinates. Errorbars indicate standard errors of the mean. Due to data redundancy in the correlation matrices underlying the scatterplots, the plot contains a kaleidoscopic pattern. Homotopic connections are excluded. The strength of the relationship indicates the level of symmetry

### Asymmetric functional connectivity

The group-mean *z* values for the RS correlation matrices were nearly all significantly larger than 0. In addition, the reproducibility of the group-mean correlation matrices across the two separate scanning days was nearly perfect (ICC_(2.1)_ of 0.998 (95% CI [0.998, 0.998])). This nearly perfect test–retest reliability between scanning days 1 and 2 indicates that there is hardly uncertainty in the established group-mean, meaning that noise can only marginally attenuate the similarity between the original and mirror RS correlation matrices. When estimating the relationship between *z* values in the mean original matrix and those of its mirror version, we found an ICC_(2.1)_ of 0.976 (95% CI [0.972, 0.980]) (Fig. 4). This means that the average connectivity of the brain during rest is for approximately 95% symmetric, and asymmetries thus accounting for only a few percent of the variance.

**Fig. 5:**
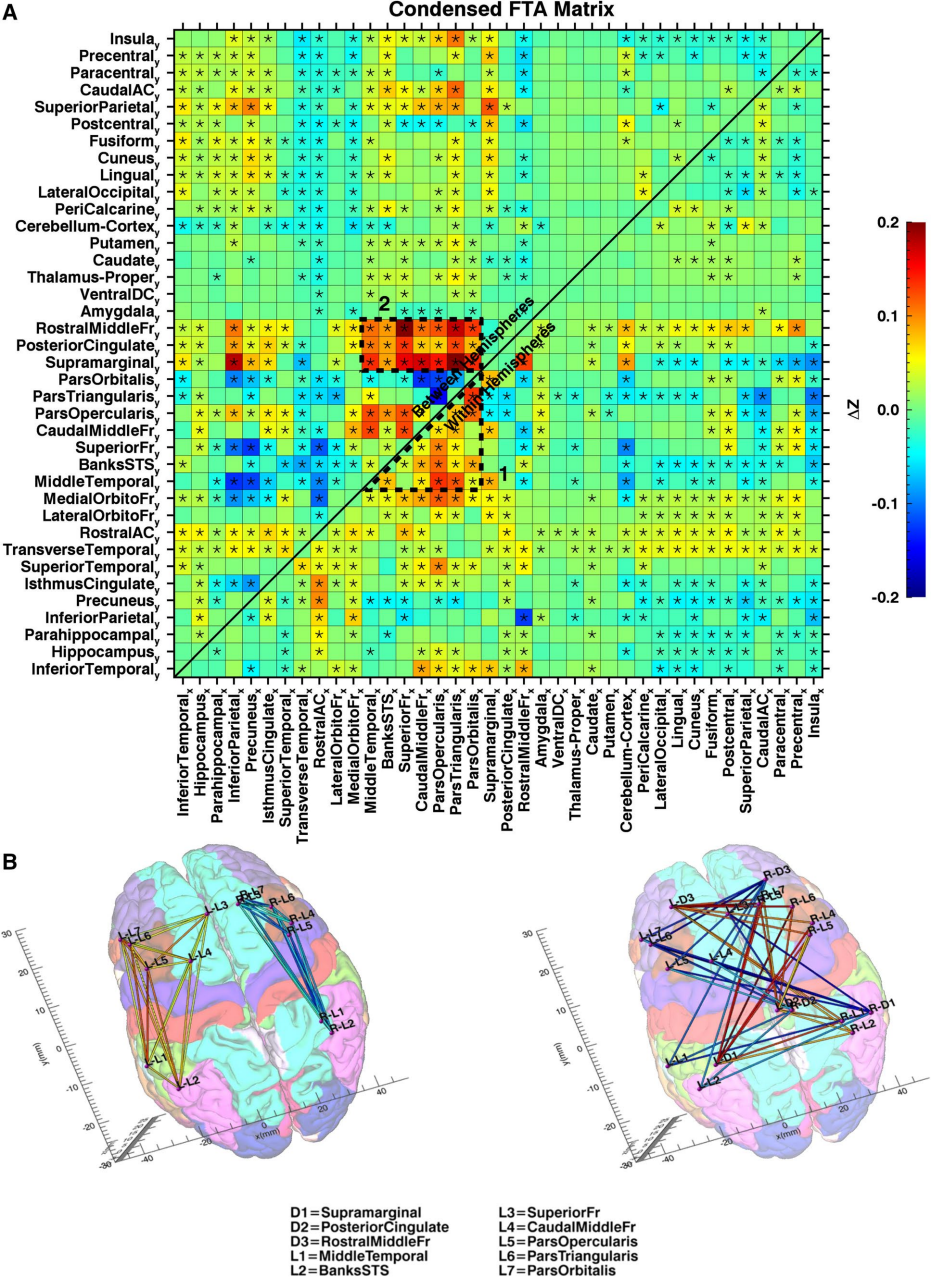
**a** Mean AFC for the connections between the different ROIs. Colors indicate the mean differences in *z* values between the original and mirror matrix. Asterisks designate asymmetries significantly different from 0 (*p* < 0.05; Bonferroni corrected). The lower right portion of the matrix shows asymmetry of within-hemisphere connections, meaning the connectivity between left area_*x*_ and left area_*y*_, minus the connectivity between right area_*x*_ and right area_*y*_. The top left portion of the matrix shows asymmetry of between-hemisphere connections, meaning the connectivity between left area_*x*_ and right area_*y*_, minus the connectivity between left area_*y*_ and right area_*x*_. The sequence of ROIs along the *x* and *y* axes was adjusted, so that ROIs with similar patterns of asymmetry are clustered together. Two clusters containing a large portion of the most pronounced asymmetries are delineated by an intermittent line. **b** Connectivity diagram representing the two clusters with the most pronounced AFC. Tags for the different brain areas are explained directly below the diagram. Colored lines between the ROIs indicate the strength of the asymmetry (original minus mirrored), with color coding according to the legend of **a**. The left image (1) shows the within-hemisphere asymmetry in connectivity between language areas; the right image (2) shows the between-hemisphere asymmetry in connectivity between language areas and the default mode network

Figure 5a shows the mean AFC matrix. Although there was significant AFC regarding a large portion of the correlations (Fig. 5a), many of the more pronounced asymmetries could be summarized relatively condensed by:

1. Higher correlations between the left hemisphere language areas (ParsTriangularis, ParsOpercularis, ParsOrbitalis, MiddleTemporal, CaudalMiddleFr, BanksSTS, SuperiorFr) than between their right hemisphere homologues (Fig. 5b).
2. Higher correlations between the areas harboring the default mode network in the left hemisphere (Supramarginal, PosteriorCingulate, RostralMiddleFr) and language homologue areas in the right hemisphere, compared to the correlation values between their respective contralateral areas (Fig. 5b).

### Correlates AFC-score

**Fig. 6:**
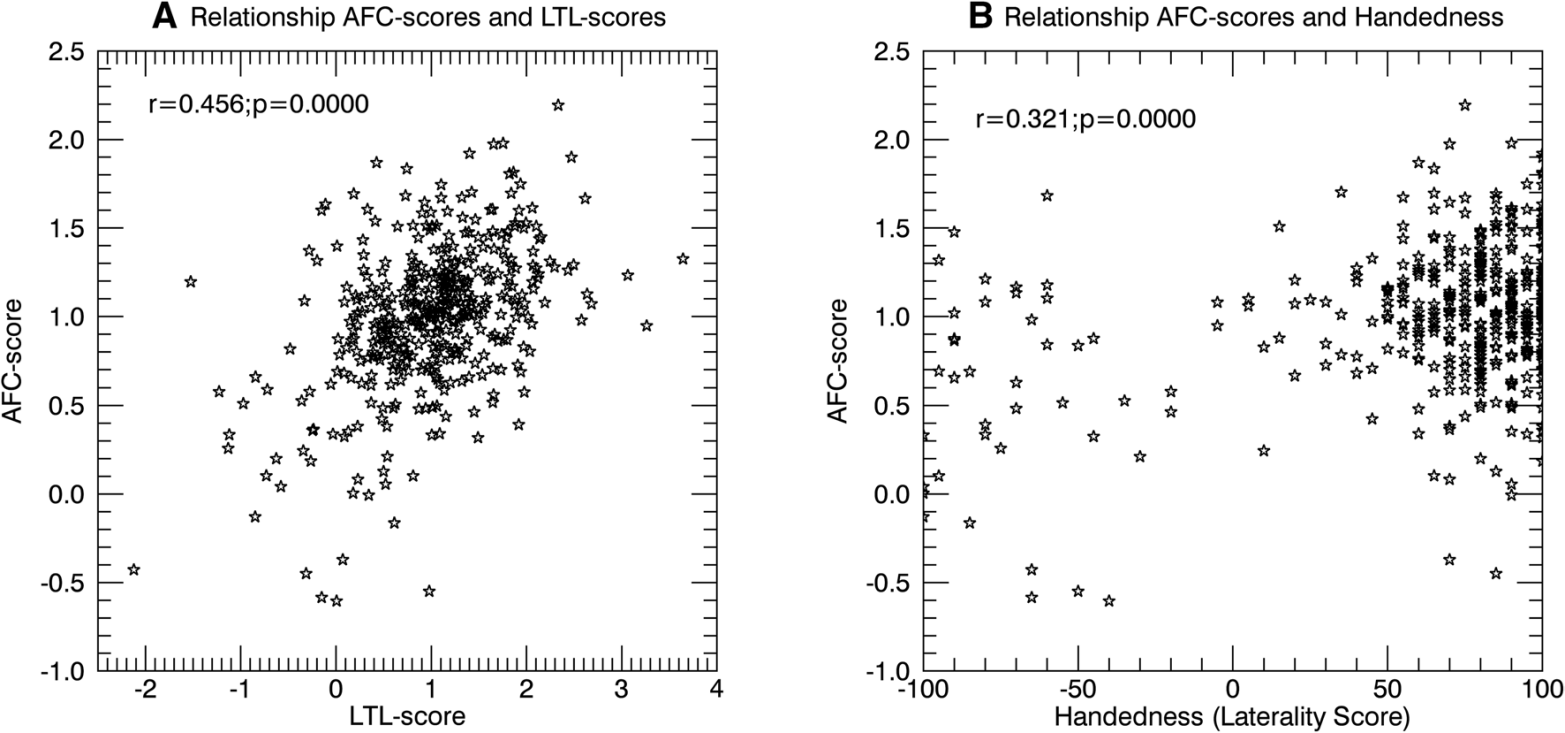
Scatterplots representing dependencies of AFC-scores. For all panels, every star represents a single subject. The different panels show. **a** Relationship between the AFC-scores and language lateralization according to the language processing task. **b** Relationship between AFC-scores and Handedness according to the Edinburgh Handedness questionnaire

AFC-score was established per subject using the values in the matrix of Fig. 5a as independent variable. The test–retest reliability of the AFC-score from day 1 to day 2 was high, but not near perfect (ICC_(2.1)_ = 0.790; 95% CI [0.752, 0.823]), meaning that the individual AFC-scores are relatively stable, and can be sensibly related to behavioral variables, but that extremely high correlations are unlikely. The AFC-score correlated positively with the LTL-score (*r* = 0.45; 95% CI [0.371, 0.522]), meaning that task language lateralization was related to AFC during rest (Fig. 6a). The AFC-score also correlated positively with handedness (*r* = 0.322; 95% CI [0.234, 0.404]) (Fig. 6b), as did LTL-score (*r* = 0.203; 95% CI [0.110, 0.292]). The results in Fig. 6 clearly show a bimodal distribution for handedness as opposed to AFC-score or LTL-score, indicating that the relationship between hemispheric specialization and handedness is not straightforward linear.

In an explorative analysis, we related AFC-score to all anatomical, behavioral, and individual difference measures as acquired by the HCP (*n* = 735), using correlations for continuous and *F* tests for categorical variables. All tests were corrected for multiple comparisons using a Bonferroni correction. A full list of the used variables with a brief description can be found at https://wiki.humanconnectome.org/display/PublicData/HCP+Data+Dictionary+Public-+500+Subject+Release. This analysis revealed further relationships between AFC-score and both left and right Cerebellar grey matter volume (*r* = 0.22 for left; *r* = 0.22 for right), and between AFC-score and visual acuity (Electronic Visual Acuity Denominator (*r* = − 0.23) (*p* < 0.05 corrected; for 735 comparisons). Note that these correlations are only just significant (critical *r* = 0.193) and that the magnitudes of these correlations are most likely inflated due to the Bonferroni correction. Handedness is thus the only HCP behavioral variable with a substantial relationship with AFC-score.

### PCA results

**Fig. 7:**
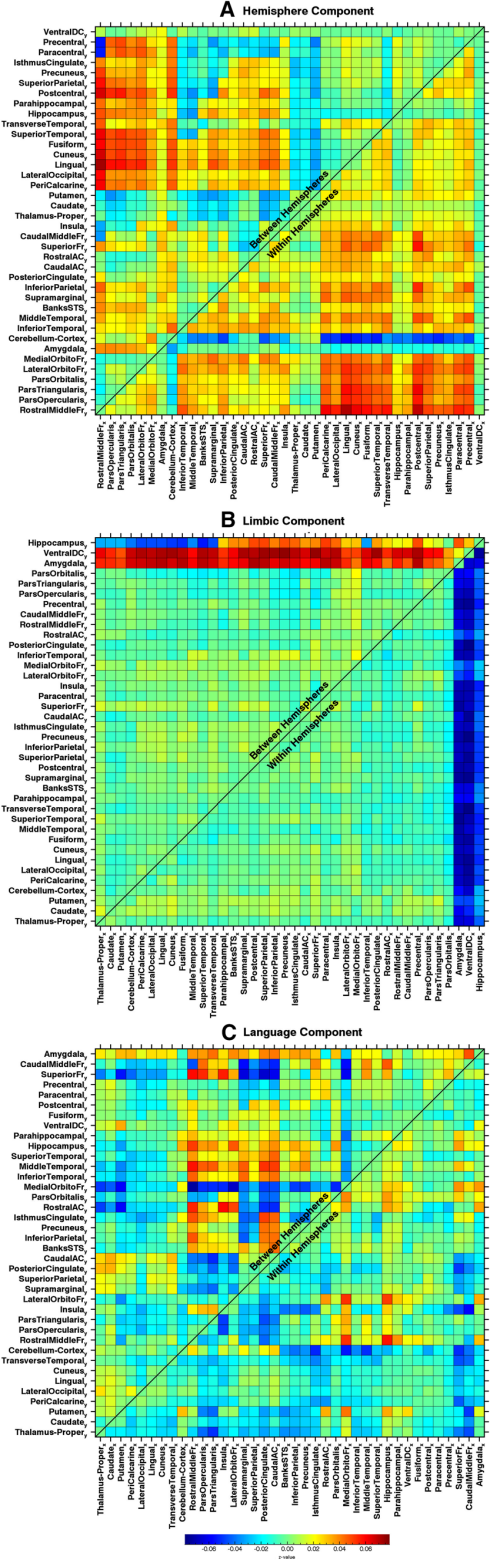
AFC matrices formed by the coefficients of the three principal components. Values in the matrices should be interpreted likewise as the ones in Fig. 5a. The components are scaled, such that the root mean square of the scores is 1. The sequence of ROIs along the *x* and *y* axes was adjusted, such that ROIs with similar patterns of asymmetry are clustered together. Note that the sign of principal components and thus also of these corresponding AFC matrices is completely arbitrary

The PCA on the AFC matrices revealed several coherent patterns, which suggests that components are actually linked to underlying brain physiology, instead of being mere statistical entities. To obtain a sense of their reliability, we repeated the PCA using the AFC matrices of only day 1 and only day 2. Then, we linked the components of the separate sessions by finding the maximum Pearson correlation with any of the components of the combined session. The correlation between the linked components reflects how well components can be reproduced in separate sessions. The three components that explained the most variance had either good or excellent reliability (Table 1) and are displayed in Fig. 7. The components were named for convenience. Note that the sign in both the figures and descriptions of the components is completely arbitrary. Also note that this sample of 3 is by no means intended to represent an exhaustive description of existing patterns of AFC, considering the rather strict criteria for selection. The three components are detailed as follows:

**Table 1:** Statistical properties of the three principal components explaining the most variance

Component^a^	Variance explained (%)^b^	Between session reliability (ICC_(2.1)_)^c^	Similarity session 1 ans 2 (*r*)^d^	Similarity session 1 (*r*)^e^	Similarity session 2 (*r*)^f^
Hemisphere	6.33	0.44	0.92	0.93	0.95
Limbic	5.09	0.48	0.92	0.93	0.95
Language	4.29	0.64	0.50	0.87	0.72

^a^Name of the component

^b^Percentage of total variance explained by the component

^c^ICC_(2.1)_ of the principal component scores between RS data of session 1 and session 2

^d^Correlation between component of session 1 and session 2

^e^Correlation between component from combined session and component from session 1

^f^Correlation between component from combined session and component from session 2

1. The ‘hemisphere’ component consisted of increased correlations within the left hemisphere vs. the right hemisphere, with the cerebellum showing the opposite effect. This effect was most pronounced for correlations between a cluster of lateral/ventral frontal areas (RostralMiddleFr, ParsOpercularis, ParsTriangularis, ParsOrbitalis,LateralOrbitoFr, Medial OrbitoFr), and remaining cortical ROIs. The same combinations of ROIs also showed most asymmetry in between-hemisphere correlations, with higher correlations between the left lateral/ventral frontal cluster and the other right cortical areas than their mirrored versions (Fig. 7a).
2. The ‘limbic’ component involved correlations with three limbic ROIs (Amygdala, Hippocampus, VentralDC), with lower correlations between these areas and all other areas in the left than in the right hemisphere. Furthermore, the correlations between these right limbic areas and the left hemisphere were higher than their mirrored versions, excluding some hippocampal correlations (Fig. 7b).
3. The ‘language’ component involved a diverse set of ROIs, but asymmetries were more pronounced for between than for within-hemisphere correlations (Fig. 7c). The component’s name was given according to its relationship with LTL-scores and AFC-scores (see further below).

### Reliability and correlates of the principal components

To establish if these three components reflected trait-like/stable characteristics of individual subjects, the scores of each component and each subject were reassessed for the 2 scanning days separately, to assess their test–retest reliability. This was done by sequentially fitting the three matrices containing the principal component coefficients (Fig. 7), to the AFC matrices of individual subjects as calculated separately for scanning days 1 and 2, using a linear regression. This resulted in a regressor coefficient (component score) for each subject and each component, for each scanning day. Subsequently, the ICCs of the scores for the three components were calculated, which are displayed in table I. We observed that for the hemispheric and the limbic component, the reliability was moderate, while the language component had good reliability.

We found that none of the behavioral and individual difference measures as assessed by the HCP correlated significantly with the score for either the hemispheric or the limbic component (*p* < 0.05; Bonferroni corrected). The score on the language component was, however, significantly correlated with AFC-score (*r* = 0.66), LTL-score (*r* = 0.27), and with handedness (*r* = 0.21), indicating that this component was a reflection of language lateralization (*p* < 0.05; Bonferroni corrected).

## Discussion

According to our metric, the mean connectivity during RS was more than 95% symmetric. We found several coherent and consistent asymmetries nonetheless. The group-mean AFC consisted foremost of (1) higher correlations between language areas in the left hemisphere than between their right hemisphere homologues, and (2) higher correlations between the default mode network in the left hemisphere and language homologue areas in the right hemisphere, than between language areas in the left hemisphere and the default mode network in the right hemisphere. The extent to which individual subjects exhibited this pattern correlated with LTL and handedness. Further exploration in intersubject variation in AFC revealed several additional asymmetries, one involving entire hemispheres, and another involving correlations with limbic areas.

The anatomical distribution of resting-state networks already suggested a high level of symmetry, and this study concretely estimates this level at 95%. This implies that effect sizes of asymmetries are modest at best, meaning that prolonged RS measurements are necessary to obtain stable estimates. Alternatively, the high level of symmetry suggests that using the mirror connections in stroke or tumor research can, in many cases, be a valid strategy.

The mean AFC indicates stronger connectivity amongst language areas as compared to amongst their contralateral homologues, which agrees with language function as one of the key lateralized features of the brain. This finding also concurs with the presence of left lateralized hubs in left hemisphere language areas during RS as was detected using a graph-theoretical approach (Nielsen et al. 2013). In addition to the asymmetries in connections within hemispheres, this study also addressed asymmetry of between-hemisphere connections. Surprisingly, these included the strongest effects which involved the interhemispheric correlations between language areas and the default mode network, with stronger correlations between right language homologue areas and left default mode network, than between their contralateral homologues. While one might have predicted language areas to interact with the default mode network, it is rather unexpected that such interaction is lateralized. This observation is, however, in line with previously observed grey matter as well as RS asymmetries in the default mode network (Saenger et al. 2012).

Note that the asymmetry in the between-hemisphere correlations for language areas and the default mode network are by no means certain to originate from asymmetries in direct white matter tracts, considering the relatively sparse heterotopic as opposed to homotopic connectivity through the Corpus Callosum (Jarbo et al. 2012). However, we currently have no conclusive alternative model explaining how this asymmetry may have arisen. While theoretically it might be that this pattern is somehow the result of intrahemispheric inhibitory white matter connections (Singh and Fawcett 2008), our findings did not show pronounced asymmetries in intrahemispheric correlations between language areas and the default mode network. This would imply that such intrahemispheric inhibitory connections would have little direct influence on BOLD connectivity, which seems unlikely. The possible role of white matter asymmetries in our findings needs further exploration, perhaps using diffusion tensor imaging.

The extent to which individual subjects exhibited the pattern of mean AFC was predictive of language lateralization, as was the language component score. Several previous studies have demonstrated a relationship between aspects of resting-state activity on one hand, and language lateralization or handedness on the other (Wang et al. 2014; Tzourio-Mazoyer et al. 2015; Joliot et al. 2016). Correlates with language or handedness include the strength of homotopic (between homologue areas) connectivity (Tzourio-Mazoyer et al. 2015), the ratio of the strength of ipsilateral and contralateral connections of voxels (Wang et al. 2014), and the patterns of asymmetries of within-hemisphere connections (Joliot et al. 2016). The latter measure comes closest to the metric employed here, but did not include asymmetries of between-hemisphere connections. The current study elaborates on these previous findings by linking language and handedness to more specific asymmetric connections, including the aforementioned interhemispheric connections between language areas, and interhemispheric connections between language areas and the default mode network.

The strength of the correlation between AFC-score and LTL-score was only moderate (*r* = 0.455), which might be caused by combined imperfect reliability of both measures. Whereas AFC-scores have good (but not perfect) reliability, the relatively short length of the language processing task (± 7.5 min) could have made the reliability of individual estimates of LTL suboptimal. A stronger relationship might have been observed if a longer language task were used. In addition, the original intent for the HCP language task was mapping semantic language processing, as opposed to more general language function as is common for the purpose of determining language lateralization. Performance of mathematical operations was used as reference condition, which may, by itself, be left lateralized (Burbaud et al. 1995; Krueger et al. 2008). The effect sizes of the LTL-scores may thus have been suboptimal, which might have attenuated the strength of the relationship between AFC-scores and LTL-scores. Nevertheless, the imperfect reliability of AFC-score even with the current state-of-the-art RS data prevents its use for determining language lateralization in individual subjects such as for presurgical mapping. The asymmetry does, however, provide a potential straightforward research tool for investigating hemispheric dominance in prelingual, as well as non-human subjects.

Handedness was the only metric of all anatomical, behavioral, and individual difference variables that had an at least moderate correlation with AFC (*r* = 0.322). Although it is generally assumed that language lateralization was predictive of hand preference (Knecht 2000), recent findings have questioned this relationship when disregarding subjects who are strongly right language lateralized (Mazoyer et al. 2014). Although our study was not specifically aimed at investigating handedness and included far more right than left-handers, we observed the relationship with handedness in spite of the absence of strongly negative AFC-scores or LTL-scores in our sample (Fig. 6), which would represent an equivalent of strongly right language lateralized subjects. In theory, one might explain this discrepancy of results by arguing that handedness as a sensorimotor phenomenon, instead of language lateralization, determines the mean AFC matrix, and thus AFC-scores. We believe this to be highly unlikely, however, considering that all correlations between LTL-score, handedness, and AFC-score were significantly positive, and that asymmetries were not overrepresented in connections with motor areas as would be predicted based on the fact that hand preference can be decoded from RS correlations between the different motor areas (Pool et al. 2015). We have as yet no explanation for the apparent contradiction in results.

The relationship between AFC-score (the extent to which the group mean is represented in individual subjects) and task language lateralization shows that language lateralization to a large extent determines the group mean, most likely because the side of the language dominant hemisphere was highly unevenly distributed within the experimental population (78% were right handed). However, there are more sources that drive AFC in individual subjects. Using PCA, we found that intersubject variation in AFC was composed of several coexisting patterns, confirming the previous findings that asymmetry in RS is not a single source phenomenon (Liu et al. 2009). The moderate reproducibility of some of the principal component scores suggests that they most likely represent individual variation in the tendency to express particular asymmetric brain states or events (Petridou et al. 2013) instead of being hard wired asymmetries.

The sources explaining the most variance in the current sample were the hemisphere component and limbic component. The hemisphere component reflected relatively enhanced connectivity within either one of the two hemispheres, in combination with the contralateral Cerebellum (Fig. 7a). This component seems to match to the recently observed differences in the amplitudes of the global/mean signal variations between the hemispheres (McAvoy et al. 2015). Between-hemisphere differences in the amplitudes of global signal variations may arise when BOLD responses in one of the two hemispheres are more synchronized than in the other, which is exactly the type of asymmetry that this component is representing. The limbic component consisted of an asymmetry in connectivity between limbic and cortical areas, both between and within hemispheres. None of these components showed a significant relationship with any of the behavioral, anatomical, or individual difference measures that were acquired by the HCP. However, future studies may attempt to establish links with more elaborate behavioral measures that are assumed to have stronger links with hemispheric asymmetries. Such links may include personality measures such as ability for divergent critical thinking (Moore et al. 2009; Santarnecchi et al. 2015), but also pathological conditions such as depression and schizophrenia (Henriques and Davidson 1991; Stephane et al. 2001; Flor-Henry et al. 2004; Frith 2005; Moore et al. 2009; Nielsen et al. 2013; Santarnecchi et al. 2015).

The method for determining the ROIs plays a key role within the approach used here. We choose a Freesurfer automatic parcellation scheme (Fischl 2004; Desikan et al. 2006), which uses geometric information derived from the individual cortical model in addition to neuroanatomical convention. First, the amount of asymmetry is inversely proportional to how well the parcellation algorithm performs at defining two anatomically homologue areas, as AFC will increase when homologue areas are ill defined. Although errors in ROI definitions must have occurred as no perfect methods for areal segmentation exist, such errors cannot account for the current observations. While substantial random errors in segmentation would have prevented us from observing any effects, systematic errors can be refuted by a simple thought experiment. If two homologue areas are defined incorrectly, or more incorrectly than others, this would affect all connections involving the homologue ROI pair, not only specific ones. Anatomical asymmetry would be reflected by crossing vertical and horizontal lines of large effects (both positive and negative) in the asymmetry matrices. None of our findings meet this criterion. Second, the choice for these ROIs roughly determines the minimum spatial scale at which the asymmetries can be detected, which more or less matches the spatial scales of the RS networks as they are found by independent component analysis (Damoiseaux et al. 2006). While more detailed asymmetries could in theory be investigated using smaller ROIs, this would increase the amount of error in the ROI definitions, and reduce the statistical power of the study.

A major potential shortcoming of this study is confounding of AFC by large-scale susceptibility effects. It has been shown that such artifacts can lead to incorrect conclusions regarding lateralization of brain functions when the phase-encoding direction is either LR or RL (Mathiak et al. 2012). These effects were counteracted in the HCP pipeline by corrections for spatial distortions (Glasser et al. 2013), and by combining data sets with LR and RL phase-encoding directions. By testing asymmetry in the effect for LR vs. LR phase-encoding directions, we found that these countermeasures were almost fully successful, except for some connections involving areas close to the nasal cavities. Perhaps, around this area, even small systematic asymmetries in anatomical structure may lead to detectable asymmetries in large-scale susceptibility effects. However, none of the detected patterns of asymmetry primarily involved connectivity with these areas.

## Conclusions

In summary, asymmetry in connectivity during RS is small but consistent, and is linked to language lateralization. There are several coexisting patterns of AFC that exist apart from language lateralization, and require further research to establish their exact nature.

## Electronic supplementary material

Below is the link to the electronic supplementary material.

Supplementary material 1 (PDF 1161 KB)
