# Supplementary material for: Knowing left from right: asymmetric functional connectivity during resting state
Source: Brain Struct Funct. 2018 Jan 4;223(4):1909–22. doi: 10.1007/s00429-017-1604-y (PMC5884915; doi:10.1007/s00429-017-1604-y)
Supplement: Supplementary file 1 — Supplementary material 1 (PDF 1161 KB) [file 429_2017_1604_MOESM1_ESM.pdf]

Supplement 1. Investigating confounding effects of asymmetric large-scale susceptibility effects:

Data were acquired with the phase encoding directions along the left-right axis. This way of acquiring the data may potentially introduce confounding large-scale susceptibility effects while testing for hemispheric asymmetries. Although the data were corrected for spatial distortions (Glasser et al. 2013), signal dropout can still cause some areas to have relatively lower signal to noise ratios and therefore lower RS correlations. As the difference in the amount of signal dropout between LR and RL acquisition is likely to vary for two homologue brain areas, either LR or RL acquisition would result in biased estimates of AFC of RS correlations. When testing for group-wise differences in the original RS correlation matrices for LR vs. RL acquisition, we indeed observed several significant effects (Figure S1). These amongst others included generally higher z-values for within right, and lower z-values for within left hemisphere correlations for LR vs. RL acquisition.

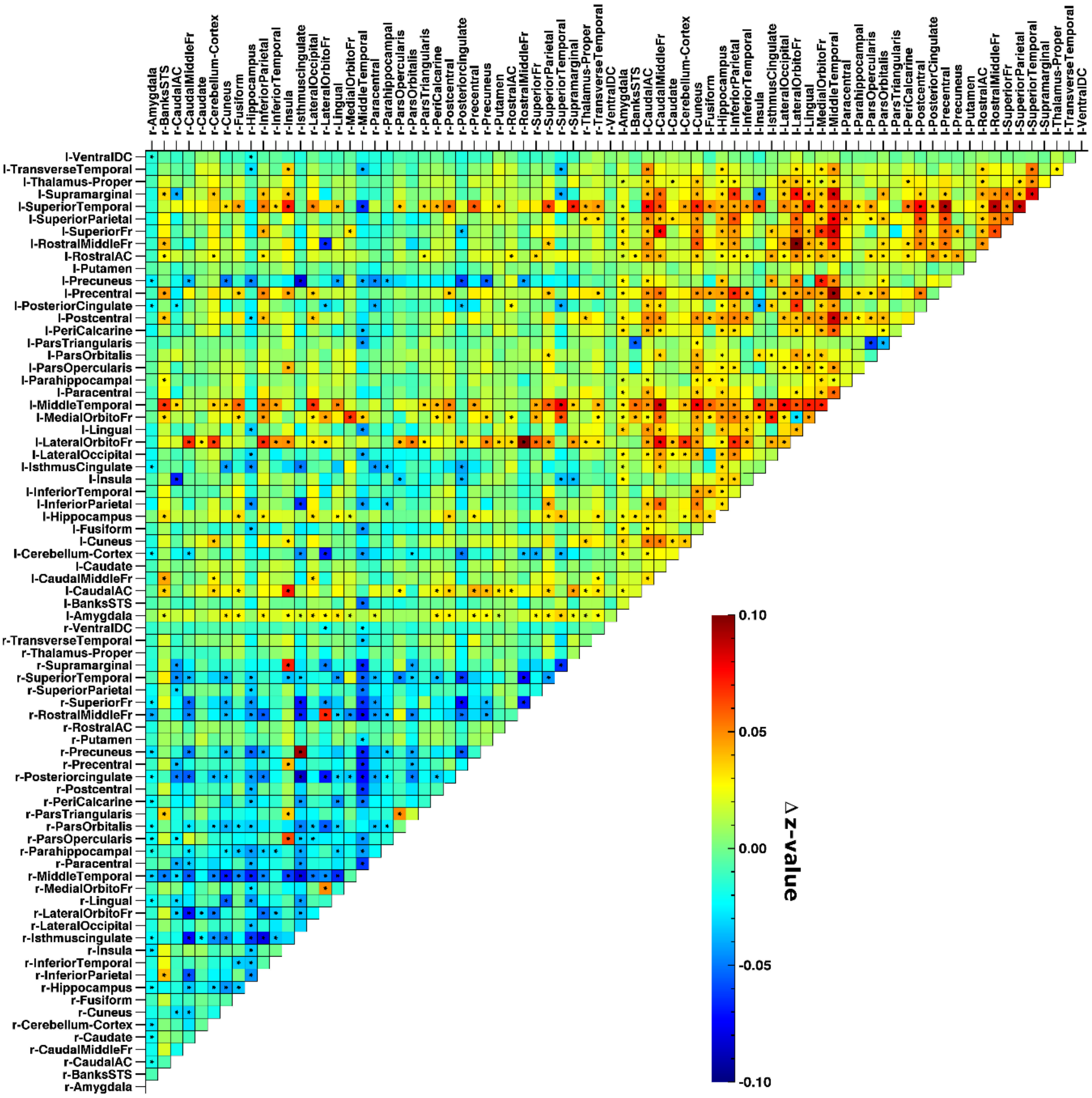

Figure S1:

Matrix representing differences in z-values between LR and RL phase encoding directions for all connections. Asterisks mark significant deviations from zero.

However, in principle these effects would cancel out when combining datasets with LR and RL acquisition, which is based on the assumption that the effects of LR vs. RL acquisition are opposite in sign for

left and right homologue brain areas. This would be reflected as an inverse correlation of the matrix with LR vs. RL effects (Figure S1) and its mirrored version, which we indeed observed (Figure S2).

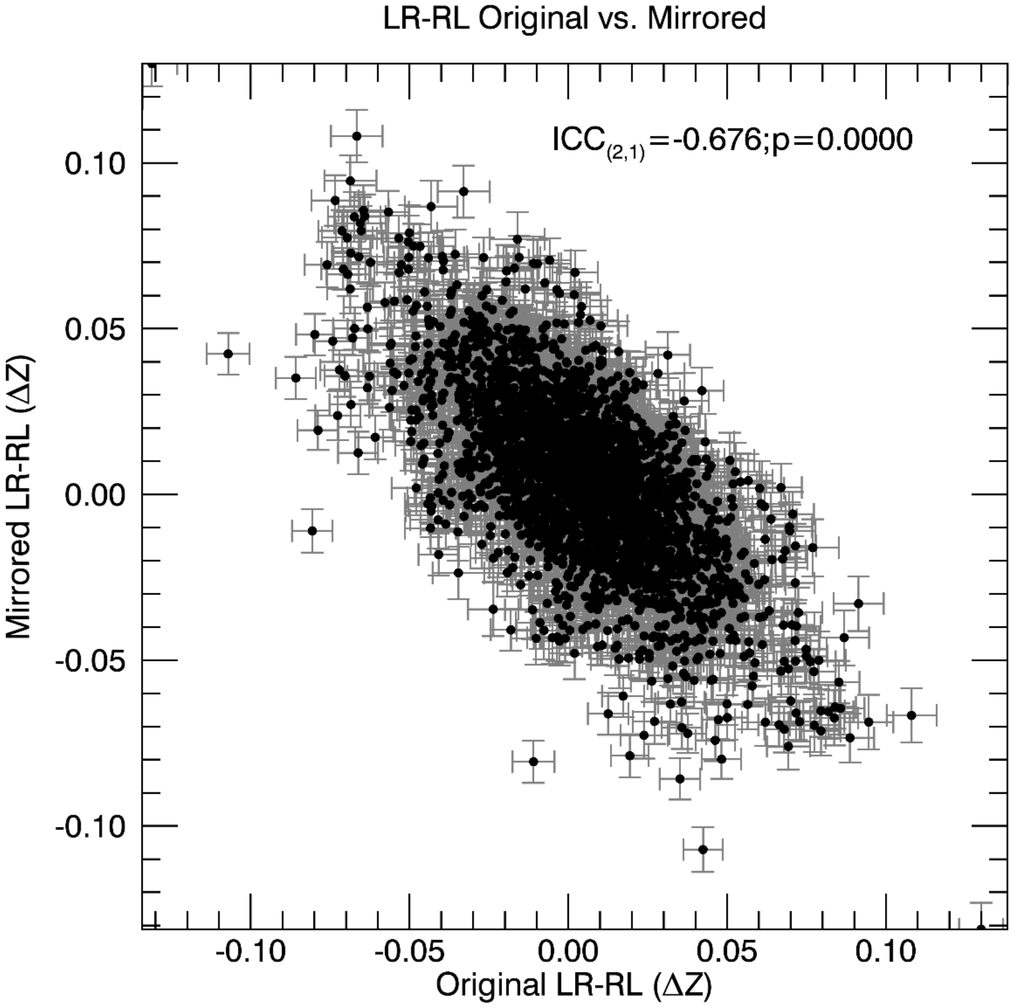

Figure S2:
Scatterplot for comparison of the group mean original and asymmetry matrices of the difference between LR and RL acquisition. In each panel, each ROI-pair is represented by a single black dot, with its value within the two compared matrices represented by its x and y coordinates. Errorbars indicate standard errors of the mean. Due to data redundancy in the correlation matrices the plot has a kaleidoscopic pattern. Homotopic connections are excluded.

To test in detail the effectiveness of the cancellation of large-scale susceptibility effects when combining the LR and RL datasets, the matrix with LR vs. RL effects (Figure S1) was added with its mirrored version, for each subject, and tested for group-wise effects. In case of perfect cancellation, this addition would result in a zero-matrix for each subject, and thus absence of significant group-wise effects. Imperfect cancellation would directly translate in AFC, and thus confound our results. Results of this analysis can be seen in figure S3, which shows only extremely small effects, of which only few were significant, and to a large extent involve correlations with ROIs close to the nasal cavity. Importantly, none of the remaining differences matched with the reported patterns of AFC of the previous analyses. It is therefore highly unlikely that phase encoding directions contributed to the results in any substantial way.

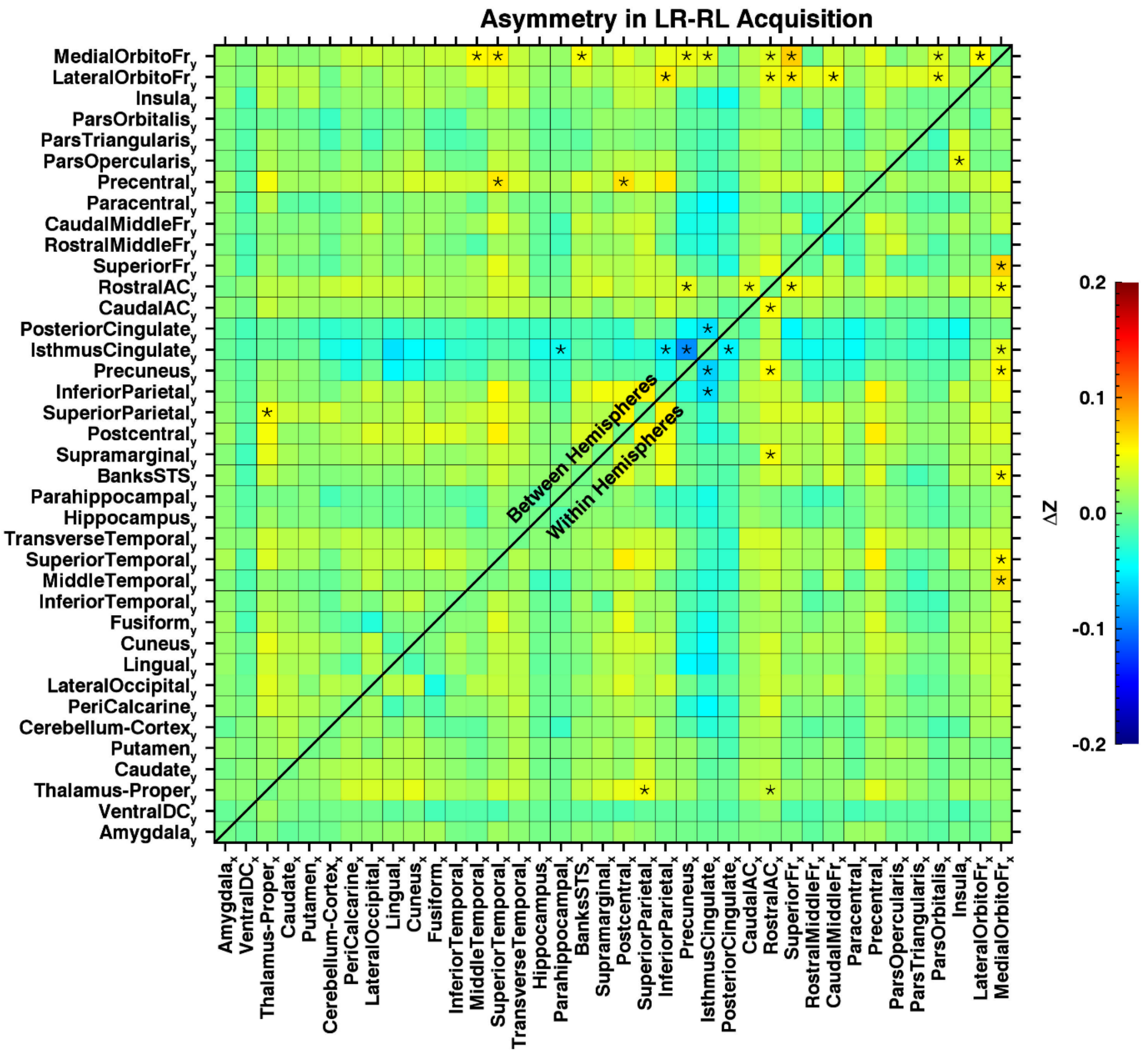

Figure S3:
Results regarding the effectiveness of the cancellation of confounding large-scale susceptibility effects when combining LR and RL acquisition. The matrix shown is the group-mean of the sum of the original and mirrored version of the correlation matrix of LR vs. RL differences (as depicted in Figure S1). Superfluous data was excluded according to the procedure explained in figure 2 in the main manuscript. Colors indicate the group-mean z-values. Values in the matrices should be interpreted likewise as the ones in figure 5A in the main manuscript.

Glasser MF, Sotiropoulos SN, Wilson JA, et al (2013) The minimal preprocessing pipelines for the Human Connectome Project. Neuroimage 80:105–24 . doi: 10.1016/j.neuroimage.2013.04.127
